# Silk Fibroin-Enriched Bioink Promotes Cell Proliferation in 3D-Bioprinted Constructs

**DOI:** 10.3390/gels10070469

**Published:** 2024-07-17

**Authors:** Sara Lipari, Pasquale Sacco, Eleonora Marsich, Ivan Donati

**Affiliations:** 1 Department of Life Sciences, University of Trieste, Via Licio Giorgieri, n.5, I-34127 Trieste, Italy; sara.lipari@phd.units.it (S.L.); psacco@units.it (P.S.); 2 Department of Medicine, Surgery and Health Sciences, University of Trieste, Piazza dell’Ospitale, n.1, I-34129 Trieste, Italy; emarsich@units.it

**Keywords:** 3D bioprinting, silk fibroin, extracellular matrix-like microenvironment

## Abstract

Three-dimensional (3D) bioprinting technology enables the controlled deposition of cells and biomaterials (i.e., bioink) to easily create complex 3D biological microenvironments. Silk fibroin (SF) has recently emerged as a compelling bioink component due to its advantageous mechanical and biological properties. This study reports on the development and optimization of a novel bioink for extrusion-based 3D bioprinting and compares different bioink formulations based on mixtures of alginate methacrylate (ALMA), gelatin and SF. The rheological parameters of the bioink were investigated to predict printability and stability, and the optimal concentration of SF was selected. The bioink containing a low amount of SF (0.002% w/V) was found to be the best formulation. Light-assisted gelation of ALMA was exploited to obtain the final hydrogel matrix. Rheological analyses showed that SF-enriched hydrogels exhibited greater elasticity than SF-free hydrogels and were more tolerant to temperature fluctuations. Finally, MG-63 cells were successfully bioprinted and their viability and proliferation over time were analyzed. The SF-enriched bioink represents an excellent biomaterial in terms of printability and allows high cell proliferation over a period of up to 3 weeks. These data confirm the possibility of using the selected formulation for the successful bioprinting of cells into extracellular matrix-like microenvironments.

## 1. Introduction

Three-dimensional (3D) bioprinting is an innovative technology that combines engineering, materials science, and biology to create intricate 3D structures [1,2]. By spatially controlling the deposition of a bioink—a mixture of polymer(s) and cells—this technique enables the creation of 3D cell-laden constructs [3,4]. In other words, bioprinting offers the advantage of providing cells with a 3D culture system that closely resembles the extracellular microenvironment, which is known to influence cell function, tissue development, and disease progression [5,6,7]. Among the different existing bioprinting methods (e.g., inkjet, laser-assisted), extrusion-based systems are characterized by their versatility in handling different bioinks, promoting the development of innovative and customized formulations that are targeted at specific goals [8,9,10]. Extrusion-based bioprinters use pneumatic or mechanical systems, such as pistons, to precisely deposit the bioink layer-by-layer and according to predefined architectural designs [2]. Regardless of the method used in bioprinting, the bioink plays a decisive role as it must show good mechanical and biological properties. It must facilitate the printing process with suitable rheological properties that ensure successful extrusion and stability after printing [11]. On the other hand, it must be cytocompatible, and, ideally, it should be degradable allowing the eventual replacement with native tissue [12]. In view of its significant potential, 3D bioprinting is driving ongoing research into the development of new biomaterials. Silk fibroin (SF) which is a natural fibrous protein extracted from cocoons of *Bombyx mori*, is emerging as a promising bioink component thanks to its biodegradability, cytocompatibility and advantageous biological activity [13,14,15]. Notably, SF has been shown to promote the multilineage differentiation of stem cells [16,17,18,19]. However, pure SF is not ideal for bioprinting, so different methods have been proposed to functionalize SF for bioprinting as the induction of SF solution gelation or the synthesis of photo-crosslinkable SF [14,20]. 

Blending SF with hydrogels made of natural polymers (e.g., alginate, gelatin, chitosan) is a promising approach to produce SF-enriched bioinks. These polymers offer proven cytocompatibility, favorable mechanical properties, and can be chemically modified to enable alternative crosslinking methods [21,22]. Alginate is a natural anionic polysaccharide derived from brown algae and is commonly used as a component of bioinks due to its high biocompatibility, minimal cytotoxicity, and low cost [23,24]. In addition, alginate can be methacrylated to produce alginate methacrylate (ALMA), which can be crosslinked by light in the presence of a photo-initiator [25]. UV light initiator systems often raise concerns about their cytotoxic effect on encapsulated cells due to the generation of reactive oxygen species (ROS) upon light exposure [26]. The visible light initiating system, composed of the photo-sensitizer eosin Y, the initiator triethanolamine (TEOA), and the catalyst 1-vinyl-2-pyrrolidinone (NVP), addresses this problem by minimizing cytotoxicity while ensuring efficient crosslinking, which is essential for achieving stable bioprinted structures [27]. Nevertheless, the highly viscous nature of alginate and ALMA causes the printed structure to spread, limiting their use as single-component bioink [28]. To overcome this limitation, alginate and its chemically modified forms are often mixed with gelatin, a protein derived from collagen [29]. The latter imparts optimal viscosity to the bioink and allows better control over the printing process due to its thermoresponsive behavior [30].

In this study, we report the development and characterization of a novel SF-enriched ALMA/gelatin-based bioink for extrusion-based 3D bioprinting. Two different SF-enriched bioinks were formulated, containing different amounts of SF but same concentrations of ALMA and gelatin. The effect of SF on the printing properties was evaluated by rheological analysis. The rheological tests also allowed an evaluation of the mechanical properties of the hydrogel which are influenced by SF. The bioink formulations were designed to contain uncrosslinked gelatin. Therefore, the stability evaluation and degradation tests aimed to investigate the release of gelatin from the hydrogels. Finally, the biological properties of SF in 2D and 3D-bioprinted cell culture models were evaluated by examining the effects on viability and growth.

## 2. Results and Discussion

### 2.1. Bioink Design and Optimization of Printability

In the present work, three different bioink formulations were prepared containing the same concentration of ALMA and gelatin but different amounts of silk fibroin (Table 1). 

**Figure 1:**
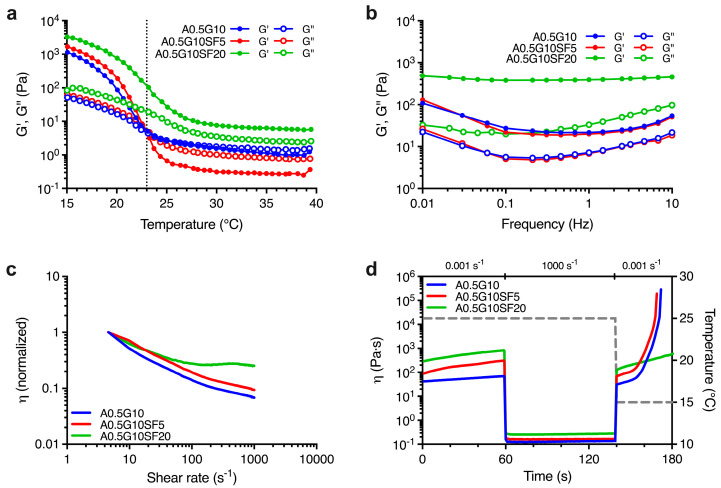
(**a**) Oscillation temperature ramp for the bioinks described in Table 1 reporting G′ and G″ moduli (Pa) vs. temperature (°C) in the temperature range *T* = 40 °C → 15 °C. (**b**) Mechanical spectra showing the dependence of G′ and G″ moduli (Pa) on frequency (Hz) at *T* = 25 °C for the formulated bioinks (Table 1). (**c**) Shear rate sweep reporting normalized viscosity vs. shear rate (s^−1^) performed on the bioinks reported in Table 1 and conducted at *T* = 25 °C. (**d**) Thixotropy tests reporting viscosity, η (Pa s), of the formulated bioinks (Table 1) and modeling their extrusion from a syringe held at *T* = 25 °C onto a print bed held at *T* = 15 °C. The first and the last steps are characterized by low shear rate (0.001 s^−1^), while the second step, simulating the extrusion, is characterized by a shear rate equal to 1000 s^−1^. Temperature variation (right Y scale) is shown by the dashed gray line.

First, we characterized the rheological profiles of the blends. To achieve this, we had to take into account that gelatin is a thermoresponsive polymer. Therefore, we first performed a series of temperature sweep experiments. We designed the experiment to investigate the viscoelastic behavior as a function of temperature ranging from *T* = 40 °C to *T* = 15 °C for all the three bioink formulations. From the results reported in Figure 1a, we can conclude that the A0.5G10 and A0.5G10SF5 bioinks exhibited a liquid-like behavior (i.e., G″ > G′) at 40 °C. As temperature decreased, the crossover point of G′ and G″ was reached (at 23 °C for A0.5G10 and A0.5G10SF5) and thereafter the bioinks began to exhibit gel-like properties (i.e., G′ > G″). Otherwise, the A0.5G10SF20 bioink retained its gel-like properties over the entire temperature range studied. To further investigate the temperature-dependent behavior of the blends, frequency sweep experiments were performed at 25 °C. For bioinks with gel-like behavior, G′ predominates over G″, while the opposite is true for viscoelastic fluids [11]. Strikingly, the frequency sweep tests revealed a gel-like behavior for all the bioinks (Figure 1b). 

The slight discrepancy between the results of the frequency and temperature sweep tests is consistent with the kinetics of the sol-gel transition of gelatin [30]. The design of the temperature sweep test (from 40 °C to 15 °C) barely delays the formation of the triple helix of gelatin. Nevertheless, frequency sweep tests confirmed a stronger gel-like behavior for A0.5G10SF20 bioink, which in turn made it unsuitable for the bioprinting process, as gel-like bioinks are usually associated with lower cell viability [31]. We then proceeded to investigate the flow properties of the bioink formulations during extrusion by shear rate sweep tests. The results shown in Figure 1c indicate that both A0.5G10 and A0.5G10SF5 bioinks exhibited shear-thinning behavior, as their initial viscosity decreased 10-fold at relatively high shear rates. In contrast, A0.5G10SF20, which had the higher SF concentration (i.e., 0.008% w/V of SF), showed very slight decrease in viscosity at low shear rates, followed by a modest shear thickening at shear rates above 100 s^−1^. In the A0.5G10 and A0.5G10SF5 bioinks, the different magnitudes of the shear-thinning effect can be attributed to the alignment of the tangled SF chains under low concentration conditions when subjected to shear stress [32]. Differently, in A0.5G10SF20, the lack of shear thinning may be attributed to the amphiphilic properties of SF, which induce the interaction with gelatin polymeric chains [33,34,35] and could eventually lead to the nozzle obstruction during the extrusion process. 

Finally, the thixotropic properties of the different formulations were studied. Thixotropy tests were developed to model the three main steps of the bioprinting process: (*i*) holding time (low shear) at 25 °C, (*ii*) extrusion (high shear) at 25 °C, and (*iii*) recovery time (low shear) at 15 °C (Figure 1d). An ideal printable bioink should have high thixotropy or self-healing ability, meaning that viscosity should drop at high shear rate and rapidly return to the original value after it is removed [36]. The A0.5G10 and A0.5G10SF5 bioinks not only recovered their original viscosity after the high-shear rate phase, but their viscosity also increased significantly when the temperature is lowered from 25 to 15 °C. This effect is attributed to the temperature-responsive properties of gelatin, as expected from temperature sweep tests [30]. In contrast, A0.5G10SF20 did not meet the printability criteria as it did not regain its original viscosity value after the high-shear rate phase. This behavior could be explained by a probable spatial hindrance on the SF chains that prevents the formation of gelatin triple helix. The rheological analysis confirmed that only formulations A0.5G10 and A0.5G10SF5 met the specifications for 3D bioprinting, in contrast to formulation A0.5G10SF20, which was not considered for further characterization.

### 2.2. Mechanical Properties and Stability Evaluation of the Hydrogels

**Figure 2:**
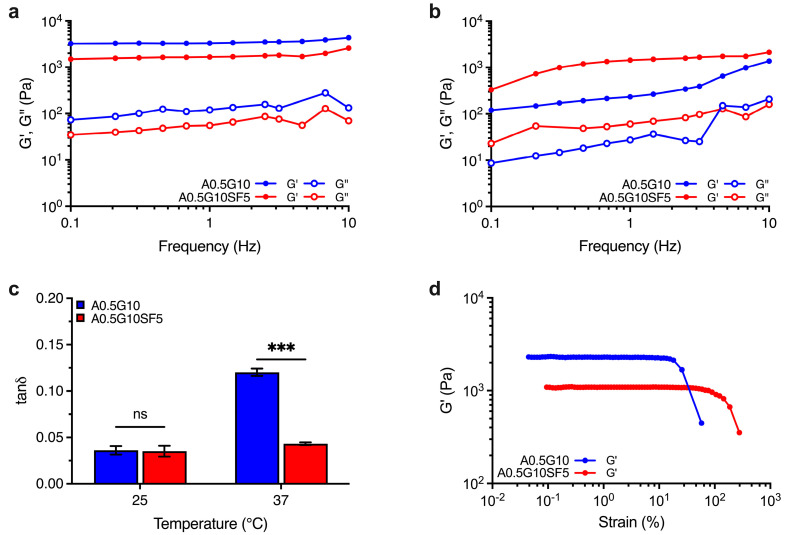
Mechanical spectra showing the dependence of G′ and G″ moduli (Pa) on frequency (Hz) for the A0.5G10 and A0.5G10SF5 hydrogels conducted at (**a**) *T* = 25 °C and (**b**) *T* = 37 °C. (**c**) Loss tangent, tanδ, values expressed as the ratio G″/G′ at frequency = 1 Hz. Data are shown as mean ± s.d. (*n* = 6). Statistics: ns = not significant; *** *p* < 0.001 (two-way ANOVA followed by Bonferroni’s Multiple Comparison post hoc test). (**d**) Elastic modulus, G′, profiles for A0.5G10 and A0.5G10SF5 hydrogels as a function on the strain (%). Measurements performed at *T* = 25 °C and at frequency = 1 Hz.

Besides printability, bioinks should offer 3D structures with suitable mechanical strength for tissue engineering applications. To this aim, we investigated the behavior of the crosslinked structures (i.e., hydrogels) generated after irradiation with visible light (λ = 400–500 nm). The mechanical spectra of A0.5G10 and A0.5G10SF5 hydrogels were recorded at both *T* = 25 °C and *T* = 37 °C, the latter to simulate biological conditions. Regardless of the SF concentration in the bioink, the mechanical spectra revealed a prevalently elastic behavior (i.e., G′ > G″) over the entire frequency range tested for both hydrogels at *T* = 25 °C (Figure 2a), resulting in relatively low values of tanδ (i.e., ratio G″/G′ at ν = 1 Hz) that are related to solid-like structures (Figure 2c). However, at *T* = 25 °C, gelatin shows the presence of triple-helical structures [30] that affects hydrogel mechanics and could mask the effect of silk fibroin. Interestingly, when the analysis is performed at 37 °C, the temperature at which the gelatin is in liquid form [30], the difference between the two samples and the effect caused by the presence of silk fibroin is more evident (Figure 2b). Comparison of the tanδ values at 37 °C revealed a greater viscous behavior of the A0.5G10 hydrogels with respect to A0.5G10SF5 hydrogels (Figure 2c), indicating a more elastic response caused by the presence of SF. Furthermore, amplitude stress sweep tests allowed us to compare the change in the elastic modulus profiles when increasing the applied stress for the two different formulations (Figure 2d). From this analysis, it was possible to observe the different linear elastic response of the two hydrogels. A0.5G10SF5 exhibits a more extended linear stress–strain response. It can therefore be concluded that hydrogels made from A0.5G10SF5 exhibit greater elasticity and resistance to temperature fluctuations. This greater elasticity can be attributed to the β-sheet generation that results in SF constructs with stronger mechanical profiles [37,38,39]. Together, these results make A0.5G10SF5 structures suitable platforms for the regeneration of elastic tissue such as bone, ligaments, or cartilage.

**Figure 3:**
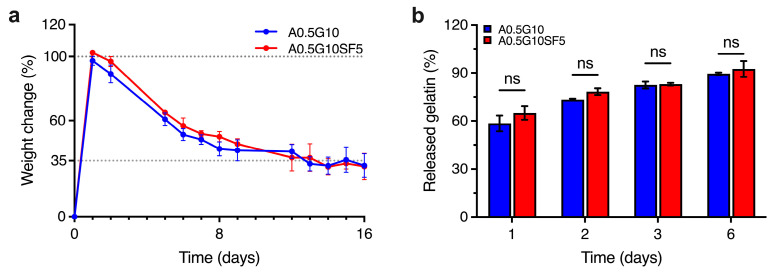
(**a**) Swelling/degradation experiments performed on the A0.5G10 and A0.5G10SF5 hydrogels (Table 1) incubated in DMEM High-Glucose cell culture medium at *T* = 37 °C and 5% CO_2_ over 16 days (*n* = 8). Data are expressed as weight change with respect to *t* = 0 (i.e., weight of dry samples). (**b**) Relative percentage of gelatin released from A0.5G10 and A0.5G10SF5 hydrogels over time measured by UV absorption. Data are shown as mean ± s.d. (*n* = 8). Statistics: ns = not significant (two-way ANOVA followed by Bonferroni’s Multiple Comparison post hoc test).

As the two formulations A0.5G10 and A0.5G10SF5 proved to be feasible in supporting the development of 3D-bioprinted systems, the next step was to evaluate the stability of the hydrogels over time. Swelling experiments performed after 16 days of incubation in cell culture medium are shown in Figure 3a. After 1 day, the medium uptake for both formulations determine a weight increase of 100% compared to the initial mass. Interestingly, the percentage of swelling decreases (i.e., loss of mass) with time, reaching a stable value of ~35% for both hydrogels from day 12 onwards. In line with the reported literature [28], a possible release of gelatin from the hydrogel matrix could explain this phenomenon. We therefore investigated the release of gelatin from the hydrogels by UV absorption at 280 nm over 6 days of incubation in PBS (Figure 3b). 

The highest release of gelatin was observed after 1 day, corresponding to 60% of the initial gelatin in the hydrogels. Moreover, gelatin is continuously released from the network until day 6, when the total gelatin released is almost 90%. The gelatin release studies parallel the mass loss observed in the swelling/degradation experiments: uncrosslinked gelatin is rapidly released and leads to an initial swelling of the ALMA network. After reaching a maximum weight gain, a loss of mass is observed in conjunction with a further loss of gelatin. From this we can conclude that from day 6 onwards, the network is gelatin-deprived. Although gelatin is released during incubation, this does not negatively affect the final performance of the 3D-bioprinted structures (Figure S4 in Supplementary Materials), since the latter can tolerate osmotic swelling.

### 2.3. Cytocompatibility Assessment and Cell Proliferation Assays

**Figure 4:**
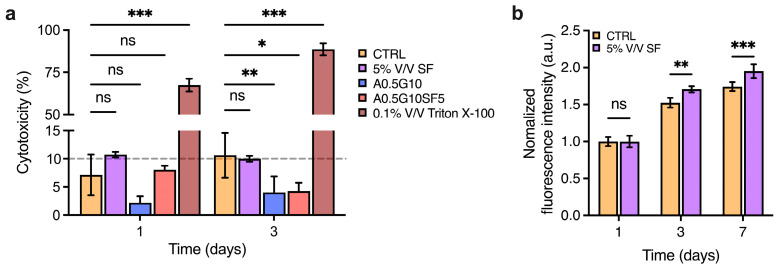
(**a**) LDH cytotoxicity assay performed after 3 days on MG-63 cells incubated with 5% V/V SF solution in DMEM High-Glucose medium and A0.5G10 and A0.5G10SF5 hydrogels. Data are compared to negative (i.e., cells in complete medium, CTRL) and positive (i.e., cells treated with 0.1% V/V Triton X-100 in DMEM High-Glucose cell culture medium) controls. Data are shown as mean ± s.d. (*n* = 8). Statistics: ns = not significant, * *p* < 0.033, ** *p* < 0.002, *** *p* < 0.001, (two-way ANOVA followed by Bonferroni’s Multiple Comparison post hoc test). (**b**) AlamarBlue assay performed at different time points (*t* = 1, 3, and 7 days) on MG-63 cultured in complete DMEM High-Glucose medium (i.e., CTRL) and in 5% V/V SF solution in DMEM High-Glucose medium. Data are shown as mean ± s.d. (*n* = 9–12). Statistics: ns = not significant, ** *p* < 0.002, *** *p* < 0.001, (two-way ANOVA followed by Bonferroni’s Multiple Comparison post hoc test).

Before proceeding to bioprint cells encapsulated in the bioinks, we wanted to assess the potentially cytotoxic effect of the SF solution and the formulated bioinks. To do so, MG-63 cells were incubated with either SF or the hydrogels for 3 days, and then a lactate dehydrogenase (LDH)-release assay was performed to assess damage to cell membranes. More specifically, the SF solution was diluted to 5% V/V in complete DMEM High-Glucose medium, and then, the release of LDH was compared to that of the control (complete DMEM High-Glucose medium, untreated). Similarly, A0.5G10 and A0.5G10SF5 hydrogels were placed over MG-63 cells and their LDH release compared to the controls. As shown in Figure 4a, results from the LDH-release assay confirm the absence of cytotoxic effect on eukaryotic cells for both SF and the hydrogels. Of note, the final concentration of SF in the medium was 0.02 mg/mL, equating to the concentration inside the bioink. Then, inspired by other studies reporting the growth-promoting properties of SF [40,41], we decided to investigate the pro-proliferative potential of SF added to the cell culture medium. An AlamarBlue assay was performed on cells incubated with SF 5% V/V after 1, 3, and 7 days of incubation (Figure 4b). Their proliferation was compared with that of cells incubated under standard conditions (i.e., complete DMEM High-Glucose medium). Strikingly, the proliferation increased from day 3. It is has been already reported that SF promotes wound healing by enhancing the proliferation of fibroblasts [42]. Xie et al. reported that SF could trigger activation of the extracellular signal-regulated kinase (ERK) signaling pathway, leading to an increase in growth [43].

**Figure 5:**
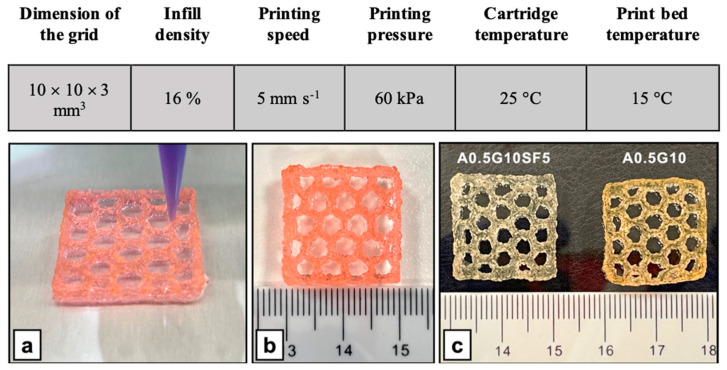
(**Upper table**) Parameters used for the 3D bioprinting of MG-63 cells using A0.5G10 and A0.5G10SF5 bioink formulations. (**Bottom panel**) Representative images of 3D-bioprinted structures (20 × 20 × 3 mm^3^, honeycomb pattern) produced with the A0.5G10SF5 bioink formulation. (**a**) Bioprinting of the 3D structure and (**b**) final result (not crosslinked). (**c**) Comparison of the A0.5G10SF5 3D structure with its SF-free counterpart after irradiation with visible light.

Given these promising results, we investigated the potential of A0.5G10 and A0.5G10SF5 bioinks for 3D bioprinting. Prior to cell encapsulation, ALMA powder was sterilized by means of UV irradiation, which causes only a slight polysaccharide chain cleavage (see Table S1 in Supporting Materials). MG-63 was then encapsulated in the bioinks at a cell density of 3 × 10^6^ cells/mL and bioprinted according to the settings described in Figure 5. Both formulations proved to be suitable to produce 3D-bioprinted structures (Figure 5). Moreover, despite its release during cell culture experiments, the role of gelatin is essential during extrusion as it allows better control over the extrusion due to its thermoresponsive behavior. The printability (Pr) index was calculated and resulted to be 1.09 ± 0.02 (*n* = 2) and 1.02 ± 0.05 (*n* = 2), respectively, for A0.5G10 and A0.5G10SF5. In an ideal scenario, Pr would equal 1, denoting pores with a perfectly square shape [44]. Therefore, the obtained results confirmed the good printability of the bioinks considered in the present work. 

**Figure 6:**
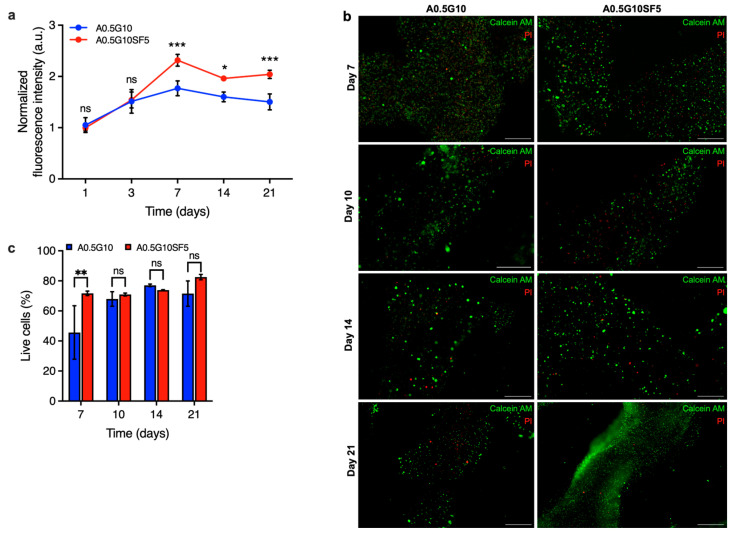
(**a**) AlamarBlue assay on MG-63 cells at different time points (*t* = 1, 3, 7, 14, and 21 days) encapsulated in A0.5G10 and A0.5G10SF5 3D-bioprinted structures. Data are shown as mean ± s.d. (*n* = 9–12). Statistics: *** *p* < 0.001, * *p* < 0.033, ns = not significant (two-way ANOVA followed by Bonferroni’s Multiple Comparison post hoc test). (**b**) Live/dead images of MG-63 encapsulated in A0.5G10 and A0.5G10SF5 3D-bioprinted structures after 7, 10, 14, and 21 days of incubation in cell culture medium (scale bar 500 µm) and (**c**) relative percentage of live cells encapsulated in A0.5G10 and A0.5G10SF5 3D-bioprinted structures at different time points (*t* = 7, 10, 14, and 21 days). Data are shown as mean ± s.d. (*n* = 2–4). Statistics: ns = not significant, ** *p* < 0.002 (two-way ANOVA followed by Bonferroni’s Multiple Comparison post hoc test).

Finally, the proliferation of cells encapsulated in the two different bioinks was examined using the AlamarBlue assay after 1, 3, 7, 14, and 21 days. As shown in Figure 6a, the proliferation rate of cells in A0.5G10SF5 hydrogels was significantly higher than those in SF-free hydrogels. These results indicate that SF is capable of exerting its effect to promote cell growth in 3D culture systems. 

Moreover, it suggests that SF is still present in biologically active concentrations despite the loss of gelatin in the hydrogels. To confirm the results of the proliferation test, further live/dead experiments were performed on the different 3D-bioprinted structures at different time points (*t* = 7, 10, 14, and 21 days) (Figure 6b). A significant difference on day 7 confirmed the effect of silk fibroin also in terms of viability, validating the results of the proliferation test (Figure 6c). These results emphasize that silk fibroin not only improves the printability and mechanical properties of the bioink, which are considered crucial for cellular processes, but also the viability and long-term proliferation of the cells. This makes our SF-enriched bioink a viable option to produce intricate structures that can easily be used in regenerative medicine or for diagnostic purposes.

## 3. Conclusions

In this study, we have reported the formulation and optimization of a novel silk fibroin (SF)-enriched bioink, which is characterized by suitable printability and mechanical properties and can promote cell proliferation. First, we tuned the SF concentration in the bioink. We tested two different concentrations of SF in the bioink (0.002–0.008% w/V) and compared them with bioinks prepared without SF. We report that silk fibroin has an impact on the mechanical properties of the bioink, as the higher concentration of SF affected the rheological profile of the gels, resulting in the impossibility to three-dimensionally bioprint it. However, the lower concentration improved the properties compared to those of the SF-free bioink. Both bioinks (i.e., A0.5G10 and A0.5G10SF5) exhibited both shear-thinning and thixotropic behaviors and were further characterized. The addition of silk fibroin proved to be strategically important to improve the printability of the bioink. In particular, the A0.5G10SF5 bioink has adequate viscosity, which is important for uniform encapsulation of the cells. In addition, the presence of SF imparts greater elasticity to the crosslinked hydrogels. Once bioink printability of the bioink and the mechanical properties of the hydrogel were established, we investigated the swelling and degradation profiles of the crosslinked hydrogels. The swelling data, consistent with the in vitro degradation, showed that there is a significant release of gelatin from the constructs within the first few days of incubation. In the bioink design, the gelatin is not crosslinked and is released from the hydrogels when placed at 37 °C for cell culture. However, the gelatin was not removed from the bioink as it serves a fundamental purpose: to enable bioprinting of the structure and prevent its collapse. The effect of SF is not limited to its rheological properties: analysis carried out on cell-laden hydrogels have shown that SF has a significant effect on cell proliferation even at low concentrations. These results demonstrate the potential use of this bioink to produce long-term 3D cell culture models, particularly for the generation of tissues such as bone and cartilage. Our findings allow further research into the direct generation of bone and cartilage tissue, possibly using stem cells. In addition, it should be investigated whether the pro-proliferative effect could be harnessed to mitigate the damage caused by bioprinting, which often leads to reduced cell viability.

## 4. Materials and Methods

### 4.1. Materials

Alginate from *L. hyperborea* (F_G_ = 0.69; F_GG_ = 0.56; weight average molecular weight (Mw) = 114,000; PI = 1.944) was kindly provided by FMC BioPolymer AS (Sandvika, Norway). Methacrylic anhydride was purchased from Sigma-Aldrich, St Louis, MO, USA. *Bombyx mori* cocoons were kindly provided by Nanodent S.r.L. (Ancona, Italy). Gelatin type B from bovine skin, bovine serum albumin (BSA), 1-vinyl-2-pyrrolidinone, eosin Y, HEPES, Lactate Dehydrogenase (LDH) Cytotoxicity Kit (TOX-7), lithium bromide, LIVE/DEAD Cell Double Staining Kit, mannitol, phosphate-buffered saline (PBS), sodium hydroxide, sodium carbonate, triethanolamine, Triton X-100, AlamarBlue reagent, Bradford reagent were all purchased from Sigma-Aldrich, St Louis, MO, USA. DMEM (Dulbecco’s Modified Eagle’s Medium) High-Glucose medium, Fetal Bovine Serum (FBS), streptomycin, penicillin, and trypsin were from EuroClone (Pero, Italy). 

### 4.2. Synthesis of Alginate Methacrylate (ALMA)

Sodium alginate (15 g) was dissolved in deionized water (375 mL). A solution of NaOH (15 g) dissolved in deionized water (75 mL) was added followed by the dropwise addition of methacrylic anhydride (120 mL). The slurry was vigorously stirred for 60 min and then added of aqueous NaOH 5 M (150 mL). The product was precipitated by addition of methanol (600 mL). The product was filtered and washed with mixtures of methanol and water with increasing amounts of methanol (60% methanol/40% water, 70% methanol/30% water, 80% methanol/20% water). The product was washed twice with each mixture (500 mL for each washing) and was filtered after each washing. Finally, the product was washed twice with methanol (500 mL), filtered, and dried at 60 °C under reduced pressure. The final yield was ~13 g (i.e., 87%). The degree of substitution was 6.7% as determined by ^1^H-NMR.

### 4.3. ^1^H-NMR Analysis

Prior to the analysis, ALMA (50 mg) was dissolved in 60 mL of deionized water. The pH was adjusted to 5.6 and the solution was kept at 90 °C for 1 h. The solution was cooled, the pH adjusted to 3.8 and the solution kept at 90 °C for 45 min. The solution was cooled, the pH was increased to reach the range 6.8–7.2 and freeze dried. ^1^H-NMR spectrum of depolymerized ALMA was recorded in D_2_O at 85 °C with a 400 VNMRS Varian (9.4 T) NMR spectrometer operating at 400 MHz. Chemical shifts are expressed in parts per million (ppm) downfield from the signal for 3-(trimethylsilyl)propane-sulfonate (see Figure S1 in Supplementary Materials).

### 4.4. Intrinsic Viscosity Measurements

Intrinsic viscosity was measured at 20 °C by means of a Schott-Geräte AVS/G automatic measuring apparatus and an Ubbelohde-type capillary viscometer (see Table S1 in Supplementary Materials) [45]. An aqueous solution containing NaCl at a concentration 0.1 M was used as solvent. The intrinsic viscosity [η] values were determined by analyzing the polymer concentration dependence of the reduced specific viscosity (*η*_sp_/*c*) and of the reduced logarithm of the relative viscosity (*ln*(*η*_rel_)/*c*) using the Huggins (1) and Kraemer (2) equations, respectively:

(1) $$\frac{\eta_{sp}}{c}=\left[\eta \right]+k^{\prime {\left[\eta \right]}^{2}}c$$

(2) $$\frac{ln\left(\eta_{rel}\right)}{c}=\left[\eta \right]-k\prime \prime {\left[\eta \right]}^{2}c$$

where *k*′ and *k*″ are the Huggins and Kraemer constants, respectively. For each polymer concentration, five replicates were averaged.

### 4.5. Preparation of Silk Fibroin (SF) Aqueous Solution

Degummed (sericin free) silk fibroin (SF) aqueous solution was prepared according to an established protocol (see Figure S2 in Supplementary Materials) [17]. Briefly, *B. mori* cocoons were cut into small pieces to increase the surface area for the degumming process. These cocoon pieces were boiled in an aqueous solution of 0.02 M Na_2_CO_3_ for 30 min. The SF fibers were thoroughly rinsed several times with deionized water to ensure that all sericin residues were completely removed. The extracted SF fibers were then dried overnight at *T* = 40 °C. After drying, the fibers were dissolved in a 9.3 M LiBr solution at *T* = 60 °C for 3 h. After dissolution, the resulting SF solution was dialyzed against deionized water at a temperature of *T* = 10 °C. During dialysis, the deionized water was changed three times a day until the conductivity of the solution reached 3 µS/cm, indicating that the LiBr and other impurities had been effectively removed. To determine the final concentration of the aqueous SF solution, a Bradford assay was performed. A calibration curve was used, which was prepared with bovine serum albumin (BSA) at known concentrations ranging from 0.1 to 2 mg/mL. By comparing the absorbance values of the SF solution with the calibration curve, the concentration of the SF solution was calculated to be 0.4 mg/mL (see Figure S3 in Supplementary Materials). Finally, the prepared SF solution was stored at a temperature of −20 °C until it was needed for further use.

### 4.6. Preparation of ALMA/Gelatin/SF Solutions and Hydrogels

Bioink solutions of alginate methacrylate (ALMA), gelatin, and aqueous silk fibroin solution (SF) were prepared as follows. For each formulated bioink solution, ALMA (50 mg) was dissolved in deionized water containing 0.01 M HEPES (pH 7.4) and 0.15 M mannitol (pH 7). For the preparation of SF-enriched bioink solutions, the SF aqueous solution was added to ALMA solution, replacing a corresponding volume of water, to obtain the final concentrations in the range 0.002–0.008% w/V (i.e., 0.02–0.08 mg/mL) of SF aqueous solution. The visible light initiator used in this work consists of the photo-sensitizer eosin Y, the initiator triethanolamine (TEOA), and the catalyst 1-vinyl-2-pyrrolidinone (NVP). Upon complete dissolution of the ALMA/SF solution at room temperature, the crosslinking mixture was added in the dark obtaining a final concentration of 5.7 × 10^−5^ M eosin Y, 0.02 M TEOA (pH 8), and 0.067 M NVP. Similarly, gelatin was dissolved in deionized water containing 0.01 M HEPES (pH 7.4), 0.15 M mannitol (pH 7), and 0.03 M NaOH at *T* = 40 °C to obtain a final concentration of 25% w/V. After its complete dissolution, gelatin (25% w/V) was added to the different ALMA/SF/crosslinker bioink solutions to obtain a final concentration of 10% w/V of gelatin for each formulated bioink. To homogenize all the added components, the different bioinks were stirred at 37 °C for 10 min. Three different bioinks were designed and the final concentrations of each component were ALMA 0.5% w/V, gelatin 10% w/V, and SF aqueous solution 0–0.008 w/V (Table 1). A more detailed composition of the three bioinks is reported in Table S2 in the Supplementary Materials. For clarity, the formulated bioink solutions are hereafter referred to as A0.5G10 (without SF aqueous solution), A0.5G10SF5 (5% V/V equal to 0.002% w/V SF aqueous solution), and A0.5G10SF20 (20% V/V equal to 0.008% w/V SF aqueous solution).

Hydrogels from the different bioink solutions were obtained upon irradiation with visible light (λ = 400–500 nm, 3 M ESPE Curing Light 2500, 230 V, 50/60 Hz) for 15 s for each cm^2^ of sample surface at room temperature to allow for photo-crosslinking, which caused a color change in the solution from a reddish hue to a light-yellow color.

### 4.7. Mechanical Characterization

The rheological properties of the different cell-free bioink formulations were evaluated before crosslinking to assess their printability and after crosslinking (i.e., hydrogels) to evaluate the mechanical properties of the final 3D constructs. All rheological tests were performed using a HAAKE MARS III rheometer (Thermo Scientific) operating under oscillatory and steady-state shear conditions. Samples were tested using the following experimental settings: titanium plates with 2° cone/plate geometry (Ø = 35 mm) and cross-hatched parallel plate configuration (HPP20: Ø = 20 mm), respectively, for uncrosslinked and crosslinked samples. A glass bell (solvent trap) was used for all tests to improve thermal control and avoid water evaporation from the samples.

To determine the sol–gel transition temperature (i.e., *T_sol-gel_*), oscillation temperature ramp tests were carried out on the bioink formulations (Table 1) in the range of *T* = 40 °C → 15 °C, with the frequency kept constant at 1 Hz. The transition temperature was determined from the crossover point between the storage modulus (G′) and the loss modulus (G″) [46]. The flow properties of the uncrosslinked bioink formulations during extrusion were then investigated by shear rate sweep measurements at *T* = 25 °C and with increasing shear rate from 1 to 1000 s^−1^. The printability of the formulations was also investigated using thixotropy tests in which both temperature and shear rate were modulated, and viscosity was measured. First, a constant shear rate (0.001 s^−1^) was applied at *T* = 25 °C for 60 s, then the shear rate was changed to 1000 s^−1^ and the temperature was held at *T* = 25 °C for 80 s. At the end of this step, the shear rate was reset to 0.001 s^−1^ and the temperature was changed to *T* = 15 °C for the following 30 s. The mechanical spectra of the uncrosslinked bioink formulations were determined by frequency sweep tests at *T* = 25 °C. The storage moduli (G′) and loss moduli (G″) were measured in the frequency range of 0.1–100 Hz while a constant stress (τ = 1 Pa) was maintained. 

For the rheological tests of hydrogels, 3 mL of A0.5G10 and A0.5G10SF5 bioink solutions (Table 1) were poured into a 6-well plate and the photo-crosslinking was performed by irradiating them with visible light. The different samples were then cut into regular cylinders using a disposable biopsy punch (Ø = 22 mm; h = ~2.8 mm). The mechanical spectra of the hydrogels were determined by frequency sweep tests performed at both *T* = 25 °C and *T* = 37 °C. The storage (G′) and loss moduli (G″) were measured in the frequency range of 0.1–10 Hz, maintaining a constant stress of 1 Pa. The extension of the linear viscoelastic region was determined by amplitude (long stress) sweep tests carried out at *T* = 25 °C using a constant frequency of 1 Hz in the stress range 0.1–1000 Pa. All the rheological tests were performed at least in triplicates. 

### 4.8. Stability Evaluation

Swelling tests were performed on cell-free hydrogels to investigate their stability as a function of time. In brief, A0.5G10 and A0.5G10SF5 bioink formulations were prepared as previously described (Table 1). Three mL of each bioink formulation was poured into a 35 mm Petri dish and crosslinked by visible light. Cell-free hydrogel disks (Ø = 10 mm, h = ~3 mm, *n* = 8) were obtained using a disposable biopsy punch and dry weighted (t = 0). Finally, the disks were immersed in DMEM High-Glucose medium (1:10 V/V) and stored at T = 37 °C. At each time point, the incubation medium was completely removed, and fresh culture medium was added after the hydrogels were weighed. The % swelling was calculated as the water uptake with respect to the initial weight via Equation (3):

(3) $$Swelling\;\left[\%\right]=\left(\frac{W_{t}}{W_{0}}-1\right)\times 100$$

where *W*_t_ is the weight of samples at different time intervals and *W*_0_ the weight of hydrogels at t = 0.

### 4.9. Gelatin-Releasing Test

Gelatin-releasing tests were performed on cell-free hydrogels to investigate their stability as a function of time. In brief, A0.5G10 and A0.5G10SF5 bioink formulations were prepared as previously described (Table 1). Three mL of each bioink formulation was poured into a 35 mm Petri dish and crosslinked by visible light. Cell-free hydrogel disks (Ø = 10 mm, h = ~3 mm, *n* = 8) were obtained using a disposable biopsy punch. Finally, the disks were immersed in PBS (1:5 V/V) and stored at *T* = 37 °C. At each time point (*t* = 1, 2, 3, and 6 days), the PBS was taken from each sample and replaced with an equal amount of fresh PBS. The absorbance of gelatin released in the PBS solution was determined using a UV spectrophotometer (Ultrospec 2100 pro, Amersham Bioscience, Amersham, UK) at 280 nm, the excitation wavelength of tryptophan. A series of dilutions of gelatin in PBS was prepared to construct a calibration curve that was used to back-calculate the concentration of gelatin released by the hydrogels. 

### 4.10. In Vitro Biological Tests and Cell Culture

For the in vitro biological analyses, the powders were UV sterilized for 30 min and the bioinks were prepared in sterile conditions. Human osteosarcoma cell line (MG-63) (ATCC CRL-1427, American Type Culture Collection, Manassas, VA, USA) was cultured in DMEM High-Glucose medium supplemented with 10% V/V fetal bovine serum, 1% 100 U/mL penicillin, and 1% 100 U/mL streptomycin (i.e., complete DMEM High-Glucose medium) at T = 37 °C and 5% CO_2_. Cell culture medium was changed every 2–3 days.

### 4.11. Cytocompatibility Assessment

The in vitro cytotoxicity of SF aqueous solution A0.5G10 and A0.5G10SF5 hydrogels to MG-63 was evaluated by assessing the release of the enzyme lactate dehydrogenase (LDH) into the cell culture medium from the cytoplasmic compartment after 1 and 3 days. The lactate dehydrogenase (LDH) cytotoxicity kit was used according to the manufacturer’s instructions. MG-63 cells were seeded at a density of 50,000 cells/well in a 24-well plate in complete DMEM High-Glucose medium. After 24 h from seeding, 0.1 mL of A0.5G10 and A0.5G10SF5 hydrogels from the different bioink formulations (Table 1) were placed in contact with the cells. In parallel, to test the cytotoxicity of SF aqueous solution, the cell culture medium was replaced with complete DMEM High-Glucose medium supplemented with a 5% V/V of SF solution (equivalent to 0.002% w/V). Cells treated with 0.1% V/V Triton X-100, untreated cells, and complete cell culture medium were used as positive controls, negative controls, and blanks, respectively.

At different time points (*t* = 1 and 3 days), a portion of the untreated samples were treated with 1/10 volume of the lysis solution and incubated for 30 min. Then, 60 µL of the medium was withdrawn from each sample to be tested, mixed 1:2 with the lactate dehydrogenase assay mixture, and transferred to a 96-well plate. After 30 min of incubation at room temperature under dark conditions, the reaction was stopped by adding 1/10 volume of 1 N HCl to each well. The absorbance was measured spectrophotometrically at a wavelength of 490 nm. The background absorbance was measured at 690 nm and this value was subtracted from the measurement of the main wavelength (490 nm). The % cytotoxicity was calculated via Equation (4):

(4) $$Cytotoxicity\;\left[\%\right]=\left[\frac{{Abs}_{A}-{Abs}_{B}}{{Abs}_{C}-{Abs}_{B}}\right]\times 100$$

where *Abs*_A_ represents the absorbance (i.e., 490 nm–690 nm) of the treated cells, *Abs*_B_ represents the absorbance (i.e., 490 nm–690 nm) of the blank (cell culture medium), and *Abs*_C_ represents the absorbance (i.e., 490 nm–690 nm) of the cells treated with lysis buffer. Two independent experiments were performed using 4 technical replicates for each condition (*n* = 8).

### 4.12. 3D Bioprinting of Cell-Laden Constructs

A suspension of MG-63 cells at a concentration of 3 × 10^6^ cells/mL was gently mixed with A0.5G10 or A0.5G10SF5 bioink solutions prepared as previously described (see Table 1 for details) using a Luer-lock syringe system at a 1:20 medium:bioink solution ratio. This careful mixing procedure ensured an even distribution of cells in the bioink. The cell-laden bioink was then filled into a 3 mL cartridge and inserted into the extrusion-based BIO X 3D bioprinter (CELLINK AB, Gothenburg, Sweden). A 25G nozzle was used to print the grid constructs (10 × 10 × 3 mm^3^) in a multi-well plate. The printing process was tightly controlled, with the printer operating at a speed of 5 mm/s and an extrusion pressure of 60 kPa. The printing temperature was set at *T* = 25 °C and the print bed was cooled to *T* = 15 °C to ensure optimal printing conditions and maintain the integrity of the bioprinted constructs. The bioprinted constructs were then irradiated with visible light (λ = 400–500 nm) and cultured in DMEM High-Glucose medium supplemented with 10% V/V fetal bovine serum, 1% 100 U/mL penicillin, and 1% 100 U/mL streptomycin at *T* = 37 °C and 5% CO_2_.

On 3D-bioprinted structures (*n* = 2 for each formulation), printability (Pr) index values were calculated via Equation (5):

(5) $$\mathrm{Pr}=\frac{\pi }{4}\times \frac{1}{C}=\frac{L^{2}}{16\;A}$$

in which *C* is the circularity of the enclosed pore, and *L* means perimeter and *A* the pore area. In this way, it was possible to calculate the bioink printability (Pr) based on the squareness of the pores inside the grid structure. A Pr value equal to 1 indicates a perfect square shape [36,44].

### 4.13. Proliferation and Viability Assay

For the evaluation of cell proliferation on 2D cell culture systems, MG-63 cells were seeded at a density of 20,000 cell/well in a 24-well plate in complete DMEM High-Glucose medium supplemented with 5% V/V SF aqueous solution (corresponding to 0.02 mg/mL) and in complete DMEM High-Glucose medium for the investigated and the control conditions, respectively. At selected time points (*t* = 1, 3 and 7 days), the cell culture medium was discarded and replaced with 500 µL of AlamarBlue reagent previously mixed with cell culture medium (complete DMEM High-Glucose medium) at a ratio of 1:30. After 3 h of incubation under dark conditions at *T* = 37 °C and 5% CO_2_, 150 µL of the mixture was withdrawn from each well and transferred to a black 96-well plate. Fluorescence was measured (λ_ex_ = 544 nm, λ_em_ = 590 nm) using a FLUOStar Omega from BMG Labtech spectrofluorometer. The AlamarBlue/medium mixture was used as a blank. Data were normalized to day 1 and expressed as a fold-change compared to day 1. Three independent experiments were performed using 3–4 technical replicates for each condition (*n* = 9–12).

Cell proliferation and metabolic activity of MG-63 cells embedded in 3D-bioprinted constructs were assessed at different time points (*t* = 1, 3, 7, 14, and 21 days) using the AlamarBlue assay. Briefly, at selected time points, the culture medium was discarded and replaced with 1 mL of AlamarBlue reagent previously mixed with cell culture medium (complete DMEM High-Glucose medium) at a ratio of 1:30. After 3 h of incubation under dark conditions at *T* = 37 °C and 5% CO_2_, 150 µL of the mixture was withdrawn from each well and transferred to a black 96-well plate. Fluorescence was measured (λ_ex_ = 544 nm, λ_em_ = 590 nm) using a FLUOStar Omega from BMG Labtech spectrofluorometer. The AlamarBlue medium mixture was used as a blank. Data were normalized to day 1 and expressed as a fold change compared to day 1. Three independent experiments were performed using 3–4 technical replicates for each condition (*n* = 9–12).

Live/dead Cell Double Staining Kit was used to assess cell viability in 3D-bioprinted models after 7, 10, 14, and 21 days of culture. In the live/dead assay, viable cells are stained green, whereas dead cells are stained red. The 3D-bioprinted cell-laden constructs obtained using A0.5G10 and A0.5G10SF5 bioinks were washed with sterile PBS and incubated in the assay solution (1 µL propidium iodide and 2 µL calcein-AM in 1 mL sterile PBS). After 1 h of incubation at *T* = 37 °C and 5% CO_2_, the constructs were observed with a fluorescence microscope (Nikon Eclipse Ti) using two different light filters (λ_ex_ = 490 nm, λ_em_ = 515 nm for green fluorescence; λ_ex_ = 535 nm, λ_em_ = 617 nm for red fluorescence). Live/dead quantification was performed using Fiji (ImageJ, version 2.9.0) and the percentage of live cells was calculated as follows (Equation (6)):

(6) $$Viable\;cells\left[\%\right]=\frac{C_{g}}{C_{tot}}\times 100$$

where *C*_g_ represents the number of spots detected in the green channel indicating the viable cells, and *C*_tot_ is the total number of green and red cells in the image. One/two independent experiments were performed using 2 technical replicates (*n* = 2–4).

### 4.14. Statical Analysis

Statistical analysis and graph elaboration were performed using GraphPad Prism 10 software (GraphPad Software, San Diego, CA, USA). Two-way ANOVA (analysis of variance) followed by Bonferroni’s multiple comparison post hoc tests were performed to evaluate differences among groups and control. Differences were considered significant for *p*-values < 0.05 (ns = *p*-value ≥ 0.05; * = *p*-value ≤ 0.05; ** = *p*-value ≤ 0.01; *** = *p*-value ≤ 0.001; **** = *p*-value ≤ 0.0001). The cartoons were created with BioRender.com.

## Figures and Tables

**Table 1:** Different composition of the bioink formulations. The final concentrations of the components are the following: ALMA, 0.5% w/V; gelatin, 10% w/V; silk fibroin, 0.002–0.008% w/V.

Sample	ALMA (w/V)	Gelatin (w/V)	Silk Fibroin (w/V)
A0.5G10	0.5% w/V	10% w/V	-
A0.5G10SF5	0.5% w/V	10% w/V	0.002% w/V
A0.5G10SF20	0.5% w/V	10% w/V	0.008% w/V

## Data Availability

Raw data generated in this study are available upon reasonable request. Correspondence and requests for materials should be addressed to the corresponding author.

## Supplementary Materials

The following supporting information can be downloaded at: https://www.mdpi.com/article/10.3390/gels10070469/s1, Figure S1: ^1^H-NMR analysis of unmodified alginate from *L. hyperborea* and ALMA; Table S1: Intrinsic viscosity measurements; Figure S2: Silk fibroin (SF) extraction procedure; Figure S3: Bradford assay for the determination of SF aqueous solution concentration; Table S2: Bioink design and components; Figure S4: Images of 3D-bioprinted structures; Figure S5: Live/dead assay.
