# Supplementary material for: Silk Fibroin-Enriched Bioink Promotes Cell Proliferation in 3D-Bioprinted Constructs
Source: Gels. 2024 Jul 17;10(7):469. doi: 10.3390/gels10070469 (PMC11275288; doi:10.3390/gels10070469)
Supplement: Supplementary file 1 [file gels-10-00469-s001.zip › gels-3112575-supplementary.pdf]

Supplementary material

Silk fibroin-enriched bioink promotes cell proliferation in 3D bioprinted constructs

Sara Lipari [1], Pasquale Sacco [1], Margherita De Angelis [2], Eleonora Marsich [3], and Ivan Donati [1,*]

[1] Department of Life Sciences, University of Trieste, Via Licio Giorgieri, n.5, I-34127, Trieste, Italy; sara.lipari@phd.units.it (S.L.); psacco@units.it (P.S.); idonati@units.it (I.D.)
[2] Nanodent S.r.L., Corso Stamira, n.61, I-60122, Ancona, Italy; m.deangelis@nanodent.it
[3] Department of Medicine, Surgery and Health Sciences, University of Trieste, Piazza dell'Ospitale, n.1, I-34129, Trieste, Italy; emarsich@units.it
* Correspondence: idonati@units.it; Tel.: +39 040 558 873

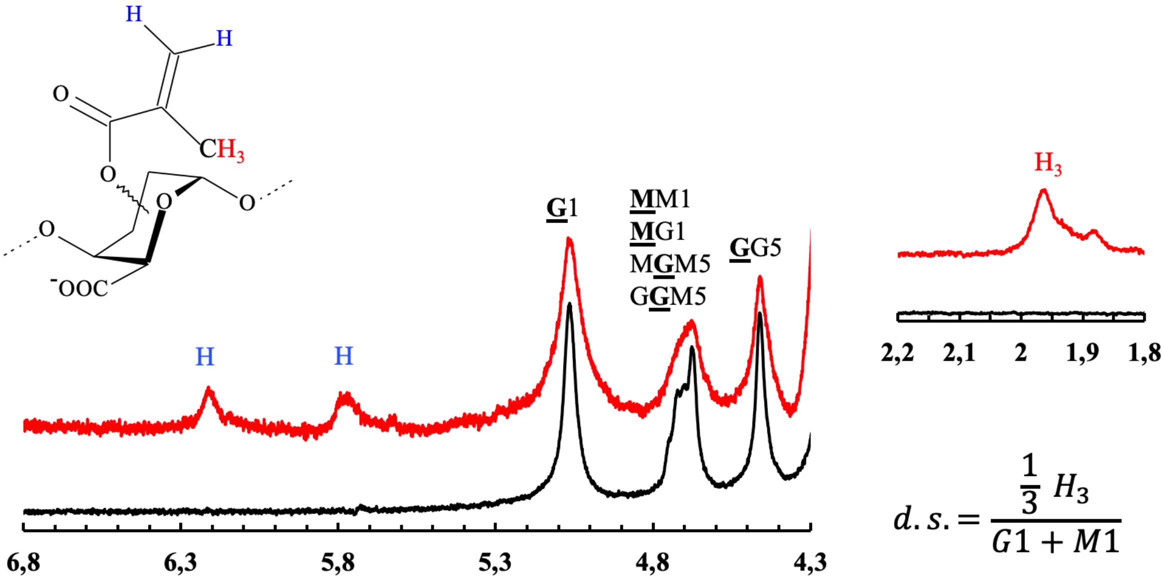

$$d.s. = \frac{\frac{1}{3}H_3}{G1 + M1}$$

Figure S1. ^{1}H-NMR analysis of unmodified alginate from *L .hyperborea* (black) and ALMA (red). Signals of the methacrylate sidechain are clearly detectable on the ^{1}H-NMR spectrum and allow to calculate the degree of substitution that is approximately 6.7 %.

Table S1. Intrinsic viscosity measurements. Intrinsic viscosity for unmodified alginate from *L. hyperborea*, ALMA and UV-sterilized ALMA, respectively.

**Sample**	[η] (mL/g)[a]
L. hyperborea	599.9±2.8
ALMA	592.4±1.3
UV-sterilized ALMA	560.9±0.6

[a]Values are reported as mean ± s.d. of the intrinsic viscosities determined using the Huggins and Kraemer equations, respectively. Solvent: NaCl 0.1M. T = 20 °C.

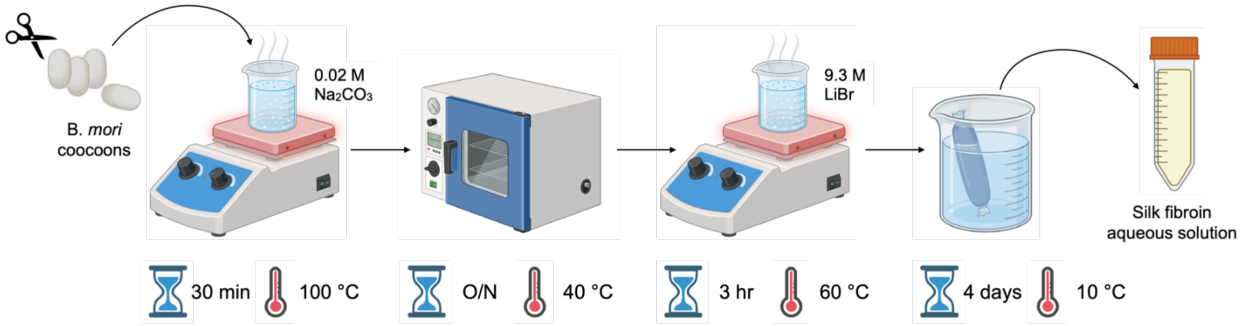

Figure S2. Silk fibroin (SF) extraction procedure. Schematic representation of the silk fibroin (SF) extraction procedure. The cocoons were cut into small pieces and boiled for 30 min in an aqueous solution of 0.02 M Na_2CO_3 to extract the fibroin fibers. The protein fibers were then collected by drying at T = 40 °C for 24 h and subsequently dissolved in 9.3 M LiBr solution at 60 °C for 3 h. This SF solution was dialyzed against deionized water at T = 10 °C for 4 days with consecutive water shifts. The final concentration of SF aqueous solution was 0.4 mg/mL.

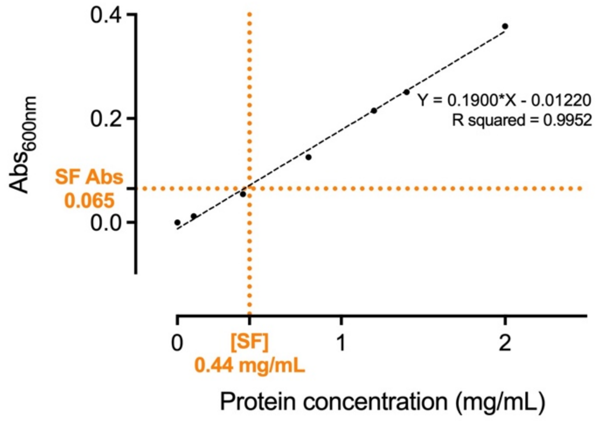

Figure S3. Bradford assay for the determination of SF aqueous solution concentration. The Bradford protein quantification assay was performed to determine the concentration of SF. A calibration curve was used with bovine serum albumin (BSA) as standard at known concentrations from 0.1 to 2 mg/mL. The absorbance at 600 nm was measured and a curve of absorbance versus protein concentration was plotted for the BSA standards and experimental datapoints were fitted with a linear equation. The related equation was then used to calculate the SF concentration based on the absorbance values.

Gelatin solution 25% w/V	
Gelatin powder type B from bovine skin	2.5 g
H_2O	7.95 mL
HEPES 1M, pH 7.4	0.1 mL
Mannitol 0.9M, pH 7	1.65 mL
NaOH 1M	0.3 mL

Bioink composition:	A0.5G10	A0.5G10SF5	A0.5G10SF20
Alginate methacrylate (ALMA)	0.05 g		
H_2O	4.6 mL	4.1 mL	2.6 mL
Silk fibroin aqueous solution (0.44 mg/mL)	/	0.5 mL	2 mL
HEPES 1M, pH 7.4	0.06 mL		
Mannitol 0.9M, pH 7	0.990 mL		
TEOA 10% w/V, pH 8	0.267 mL		
1-vinyl-2-pyrrolidinone (NVP)	0.072 mL		
Eosin Y	0.008 mL		
Gelatin solution 25% w/V	4 mL		

Table S2. Bioink design and components. (**Upper table**): detailed components of the gelatin solution (25% w/V) prepared for the bioink formulation. (**Bottom table**): detailed components of the three bioinks (*i.e.*, A0.5G10, A0.5G10SF5 and A0.5G10SF20).

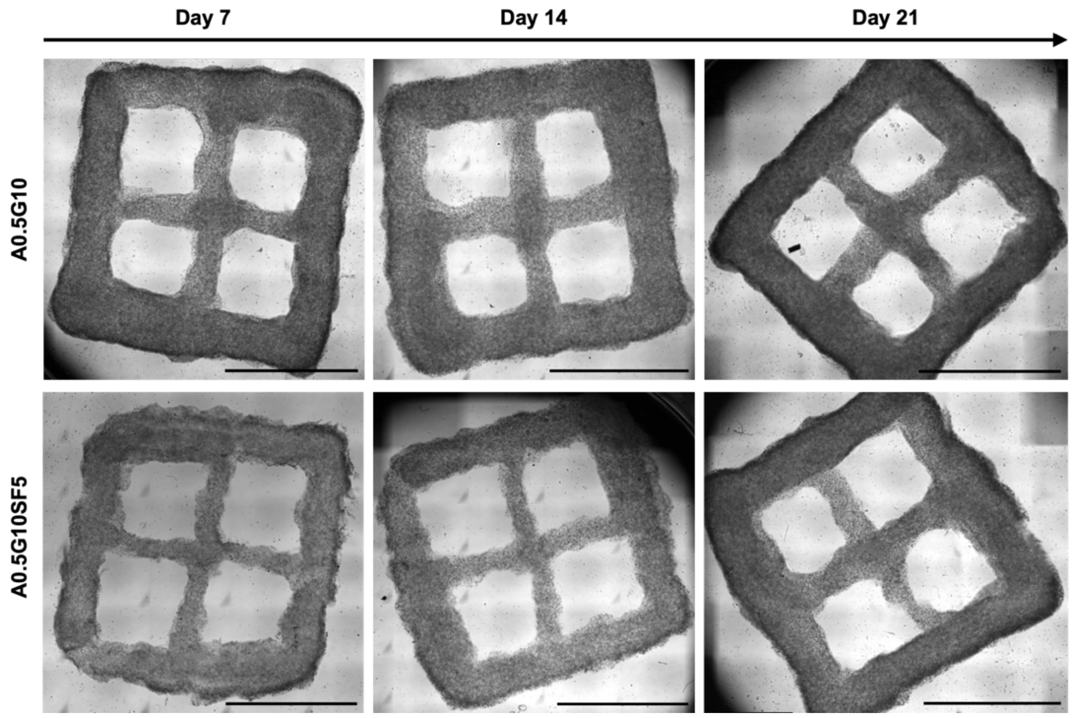

Figure S4. Images of 3D bioprinted structures. Microscopical images (4X magnification and large image setting) of A0.5G10 and A0.5G10SF5 3D bioprinted structures after 7, 14 and 21 days of incubation in DMEM cell culture medium (scale bar 0.5 cm).

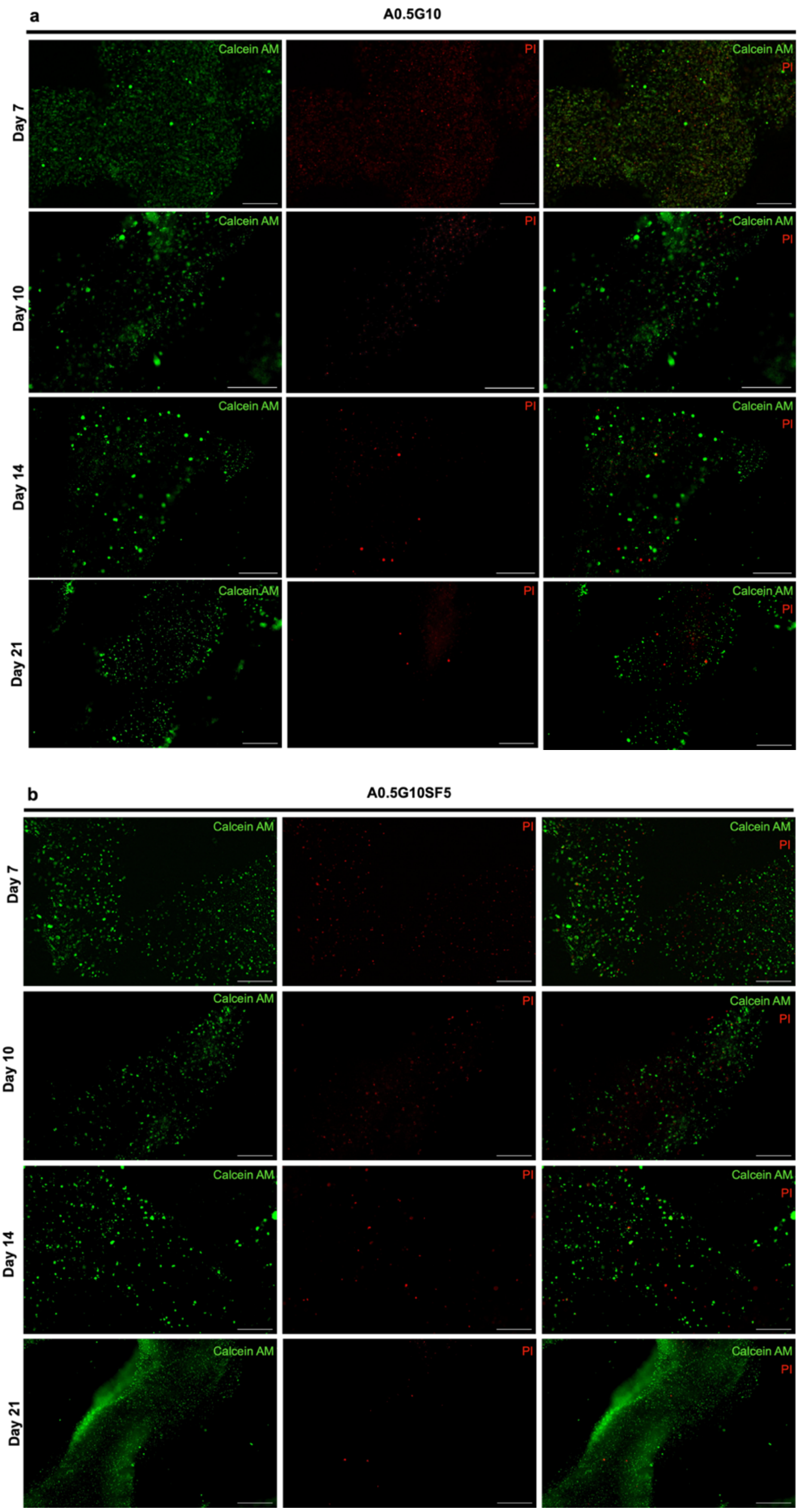

Figure S5. Live/dead assay. Live/dead images of MG-63 encapsulated in A0.5G10 (**a**) and A0.5G10SF5 (**b**) 3D bioprinted structures after 7, 10, 14 and 21 days of incubation in DMEM cell culture medium (scale bar 500 µm).
